# The Hospital. Nursing Section

**Published:** 1903-10-31

**Authors:** 


### The Hospital.

### Nursing Section.

Contributions for this Section of "The Hospital" should be addressed to the Editor, "The Hospital" Nursing Section, 28 & 29 Southampton Street, Strand, London, W.C.

No. 892.—Vol. XXXV. *SATURDAY, OCTOBER* 31, 1903.

### Notes on News from the Nursing World.

### OUR CHRISTMAS DISTRIBUTION.

It is very satisfactory to find that our readers are courteously paying attention to our request to send in contributions to our Christmas distribution of clothing without delay. Moreover, the contents of the parcels already to hand justify the hope that this year we shall be able, as we desire, to announce a record supply. In addition to the contributions acknowledged, we have also received others from Miss Bourke, Hill House, Taplow, and Miss Richardson, Harrogate. All parcels should be addressed to the Editor, 28 & 29 Southampton Street, Strand, London, W.C., and should have "Clothing Distribution" written outside them.

### QUEEN ALEXANDRA'S MILITARY NURSING SERVICE.

We are officially informed that Miss H. W. Reid has been appointed matron in Queen Alexandra's Imperial Military Nursing Service, and attached to the Connaught Hospital, Aldershot. Miss K. M. Hewetson has also been appointed staff nurse, and posted to the Royal Military College, Sandhurst.

### THE FOREIGN OFFICE AND NURSES FOR THE BALKANS.

We understand that a nurse who wrote to the Secretary of War offering her services in the event of any organisation being proposed to send nurses to Turkey, received a reply from the Foreign Office suggesting that she should apply to the Macedonian Relief Committee, of which Mr. Oliver Williams, 116 Victoria Street, S.W., is the secretary. A communication to Mr. Williams elicited an answer, dated October 26th, to the effect that at present the committee are awaiting the report of their advance party before coming to any decision as to sending nurses or doctors. Mr. Williams added that he should be glad to have full information as to qualifications and copies of testimonials in order to file them, but asked that no original testimonials should be sent. It seems improbable that the committee will determine to dispatch nurses to European Turkey.

### AMERICAN SUPERINTENDENTS IN CONFERENCE.

An extremely interesting feature of the annual Convention of the American Society of Superintendents of Training Schools this month, whose proceedings are summarised in another part of the paper by one of the members, was the number of nursing appliances shown and explained by nurses from the schools represented. Among the practical suggestions made by the authors of the various papers read was one by Miss Goodrich, who, dealing with "Points of Weakness in Hospital Construction," urged that these might often be diminished if the superintendents of nurses were more frequently consulted by those responsible for the plans of hospital buildings. Mr. Frank Miles Day, an architect of Philadelphia, subsequently treated this subject, and answered many questions which were raised by his hearers. The next Convention is to be held at Washington in January, 1905.

### THE STANDARD OF NURSING IN SWITZERLAND.

In an interesting leaflet on The Nursing Profession in Switzerland, Professor Sahli, of Berne, refers to recent efforts to raise the standard. The school for nurses founded by the Red Cross Society was opened in 1898. It is attached to a small private hospital. The training is for 18 months, six months in the mother hospital and a year in some large general hospital. The nurses receive certificates and become independent on condition that in case of war their services shall be at the disposal of the Red Cross Society. The nurse when her training is over can become a private nurse, earning from 2s. 5d. to 5s. per 24 hours; take up hospital work, or obtain one of the few posts as district nurse, earning from £40 to £48. In addition to the Red Cross Society, the "Eidgenönische gemein-nützige Frauenverein" which opened at Zurich in 1901 their school for nurses and hospital for women combined, are doing a useful work. The school undertakes to train women in various branches of nursing, the period of training being three years, part of which is spent in private nursing. We are glad to see that Professor Sahli considers the training offered under the auspices of the Red Cross Society inadequate, and suggests that it should be extended.

### ROYAL UNITED HOSPITAL, BATH.

The nurses' examinations took place at the Royal United Hospital, Bath, a few days ago. Ten third-year nurses presented themselves, and all were successful in gaining the certificate of the hospital. Nurse Helen Johnson gained the gold and Nurse Hild Drage the silver medal, both the gifts of the President, the Rev. E. Handley, M.A. Three second and six first year nurses were also examined, with most satisfactory results. Nurses Green and Shepherd, second year, won the medical books presented by Mrs. Handley; and Nurse Bartlett, first year, gained matron's prize.

### NURSES WITHOUT CAPS.

There are several features of interest about the nursing at the Wilhelmina Gasthuis in Amsterdam. The uniform of the nurses in the great municipal hospital is dark blue and a white apron with shoulder straps. Those who have passed their third year's examination are distinguished by a white brooch in the form of the Maltese Cross, but otherwise the head nurses and the juniors are similarly attired. Caps are worn by the wardmaids, but not by any of the nurses. We prefer the English custom. A feature in the long wards is an upright piano, and one of the nurses frequently sings or plays for the patients at quiet times. The nurses are on duty for eleven hours in the day, and there is an average of one nurse to four patients. It cannot therefore be said that their work is lighter than that of nurses who are employed in many hospitals supported by voluntary contributions.

### THE BRITISH GYNÆCOLOGICAL SOCIETY.

The next examination both for the maternity nursing certificate and for the gynæcological nursing certificate of the British Gynæcological Society will be held the first week in December. This will be the last examination under the year of grace offered by the Society for those nurses who have only had one year's general training and less than three months' special training. Next year candidates will be required to have at least two years' general training in addition to special training, and after 1904 the minimum standard of general training will be three years.

### NURSES FOR LINERS.

Miss Kate Penn is anxions that it should be known that she has nothing whatever to do with any movement for employing ladies who have had nursing training as stewardesses for ocean steamers. Her proposal is that there should be nurses for liners irrespective of the stewardesses. The fact that she has now received 64 applications from nurses who desire to be engaged in that capacity shows that if the shipping companies are willing to co-operate there need be no difficulty in securing the services of fully-qualified persons. Meanwhile, a nurse who actually served as a stewardess, shows, in another column, that the work might not be at all congenial to ladies with nursing training.

### THE NURSING OF INFANTS.

Some interesting details are given by Dr. John Lovett respecting the work of the nursing staff in the Thomas Morgan Rotch Memorial Hospital for Infants, at Boston, U.S.A. Dr. Lovett, who says "that most nurses not only do not know how a baby should be fed but cannot even bathe and dress one properly," is a strong advocate of training in the care of babies. At the Hospital for Infants graduate nurses only are admitted. The course is one of four months, and is divided between medical and surgical work. The mornings, for at least a month, are devoted to the out-patient department. Two weeks are spent in the food room, where the nurse has entire charge of the food. All the scrubbing and rough work is done by scrub-women or nursery maids. The instruction is by lectures by the visiting staff, recitations on the lectures, lectures by the director of nurses, and by practical teaching in the wards by the director of nurses and her assistant. Both a written and an oral examination must be passed in order to obtain a certificate. In the wards the nurses are taught, not only how to take care of sick babies, but also how to handle and take care of well babies. They receive practical instruction in the making up of cribs and beds for infants, bathing and dressing them, and in the care of the eyes, nose, mouth, ears, skin, and hair. They are also taught how to weigh babies, and how to take their temperature, pulse, and respiration. They are likewise shown what clothes a baby should wear day and night. Special attention is given to the details of the feeding of infants, to the observation and care of the napkins, and such things as the tube-feeding, irrigation of the colon, and the application of trusses are demonstrated.

### PONTYPOOL HOSPITAL.

The hospital erected on a site presented by Mr. Hanbury, of Pontypool Park, to the people of the district, embracing a population of some 40,000, was opened last week by Mrs. Hanbury, in the presence of a large company. The matron of the new institution, which is thoroughly up to date, and starts free of debt, is Miss S. A. Todd, who was trained at St. George's Hospital, and has had two years' experience as a district nurse in Cardiff, and three in a similar capacity at Retford, where she was also dispenser. She was selected out of more than 20 applicants, and she will have under her two fully-trained surgical nurses and two probationers.

### NURSES AND THEIR MEDICAL SUPERINTENDENT.

On Monday last the lady superintendent and nurses of the Mill Road Infirmary, Liverpool, prepared a pleasant surprise for the medical superintendent by presenting him with the full dress academic costume of a Doctor of Medicine in a handsome case, and a bouquet to Mrs. Nathan Raw. In making the presentation Miss Bracewell said that for some time past it had been the unanimous wish of the nursing staff to show their appreciation of the kindness which had always been shown them, both in health and sickness, by the medical superintendent, and begged him to accept the gift. Dr. Nathan Raw, before replying, donned his new robes and thanked the nurses most cordially for this spontaneous evidence of their goodwill to him, and said it would always be his endeavour to maintain the high tone of the nursing of Mill Road Infirmary. He thanked them on behalf of his wife for the bouquet.

### GRANARD NURSING QUESTION.

In reply to the offensive resolution recently passed by the Granard Guardians, Dr. Joseph Kenny, while not offering an apology which was not called for by the circumstances, has courteously made a statement that he did not intend the remotest insult to the nuns in any action he was compelled to take. It was, he added, the system of hospital management and equipment persisted in for years by the several Boards of Guardians against his wishes that he was endeavouring to reform, and to assist in terminating the matter he had no hesitation in expressing regret for the unpleasantness of the past, while he was prepared to do what he could, consistent with his personal considerations, to meet the wishes of the Guardians, especially as there was now a promise to carry out the necessary reforms.

### A DISTRICT ASSOCIATION IN THE WRONG.

The Cheltenham Board of Guardians contribute £170 a year to the Cheltenham District Nursing Association, and they have therefore a claim to be considered in the arrangements which are made in connection with the work of the nurses employed by the Association. It appears that one of the nurses has lately removed her residence from Bentham to Leckhampton, although two years ago she was allotted to Bentham by arrangement with the Guardians. As Leckhampton is six miles distant from Bentham, we are not surprised that there has been a protest on the part of the Guardians against the change. The fact that the removal took place without a word being said to them has naturally increased the irritation felt. It is urged in justification that the nurse desired to move because the people with whom she had been lodging were removing. This is not an adequate reason; and we regret that the District Nursing Association did not inform her that it was requisite for her to remain in the Bentham District. Boards of Guardians are not often so liberal in their grants to district organisations as the Guardians of Cheltenham, and at least they were entitled to be consulted in the matter before any action was taken.

### THE NURSES' TOTAL ABSTINENCE LEAGUE.

Miss Richardson, Lady Superintendent of the London Temperance Hospital, has invited the members of the Nurses' National Total Abstinence League to visit that institution on the afternoon of Saturday, November 14th, from four to six. No one will be admitted without a card of invitation, but any nurse who would like to be present can write for an invitation to the hon. secretary of the league, Miss Hilda Dillon, 47 Oakley Street, Chelsea, S.W.

### THE NURSES' HOSTEL COMPANY.

The sixth annual meeting of the Nurses' Hostel Company, Limited, was held last week. The report, which was adopted, states that the year has been an average one, "no epidemic having at any time denuded the Hostel of nurses, nor any special occasion caused a great influx of visitors." All the rooms but one in the new south block were let at the end of the financial year, and the north block has accommodated 596 visitors, paying an aggregate of 1,557 visits. The directors draw attention to the fact that the original contract for the new block has not been executed. A dividend of 3½ per cent. on the shares was declared. This absorbs £657 2s. 6d., in addition to which £105 5s. 5d. has been paid to the builders, clearing all charges on the capital account, and a balance of £166 18s. 11d. is carried forward.

### ADDITIONAL NURSES FOR WEST HAM INFIRMARY.

When a medical officer informs a Board of Guardians that the patients in the workhouse infirmary need a certain number of additional nurses, it is a very serious matter to set his opinion at defiance. If the guardians have confidence in him, their duty is to make the increase desired; if they have not, they should tell him so. An unanswerable case has been established by Dr. Greig for the appointment of more nurses at the West Ham Union Infirmary. There are, he states, 725 sick cases under his charge, the majority of which are bad cases. The number of qualified nurses in the workhouse is 13, and there are 15 temporary nurses. Dr. Greig wants the permanent staff augmented by 21, which is only, as he points out, an addition of six, although the proposal has been criticised by some of the guardians as if it had been suggested that 21 new nurses should be forthwith introduced into the wards. After a long discussion on the question at the last meeting, it was wisely decided to concede the necessary addition. We can only wonder, in the circumstances, that it was not pressed before.

### DISTRICT NURSING IN TORQUAY.

In accordance with the resolution passed at the annual meeting, the District Nurse Institution at Torquay is now a purely philanthropic institution, the private nursing staff hitherto employed having been abandoned. It has been necessary hitherto for the annual subscriptions to total at least £250, but in future nearly double that amount will be required to keep it in thorough working order. There are at present a matron and five district nurses on the staff, who paid something like 14,000 visits last year to the sick poor. A number of ladies, who are on the committee, have resolved to call upon the residents of the town with a view to impressing the needs of the institution upon them, and in the hope that more subscriptions, which are urgently required, will be forthcoming.

### PROGRESS AT CRIEFF.

The members of the Crieff and District Nursing Association may be congratulated upon the entirely satisfactory character of the report which was considered at the annual meeting. The organisation has now been established six years, and while the total number of cases nursed continues to increase, the receipts continue to be largely in excess of the disbursements. In the year ending September 30th the increase was £282 18s. 6d., and the outgoings £146 2s. Lady Ancaster, who presided at the meeting, and referred to the success achieved by the association, said that when there was a large balance to its credit, the amount was put into the Endowment Fund. The latter, it may be added, now exceeds £300.

### NEW HOME AT HORSFORTH.

There has just been opened at Horsforth, in Yorkshire, a home for the two district nurses. The ceremony was performed by Mrs. Boyd Carpenter, wife of the Bishop of Ripon, who spoke of the excellent work done by the nurses, and congratulated the Horsforth Association on having secured a home in a central position in the village. The total cost of the house and furniture was about £415. We regret to learn that the financial support given is not so considerable as it ought to be, but the development of the movement which usually follows the acquisition of a home may tend to improve the pecuniary position.

### SHORT ITEMS.

The Matrons' Council is initiating a movement for the erection of a national memorial to the nursing sisters who died while they were employed in South Africa in the service of their country.—An entertainment of a very successful character was given to the in-patients of the Cancer Hospital, Fulham Road, on Thursday evening last week, by the Rev. J. L. Geffen and friends.

### Some Practical Points in the Nursing of Diseases of the Skin.

By Norman Meachen, M.D., B.S., M.R.C.P.Lond., M.R.C.P.Edin., Physician to the Skin Department, Tottenham Hospital, N.

Cases of skin disease are not, as a rule, favourite ones among nurses. Their admission into the ward is not welcomed with the same eagerness which is shown towards a pneumonia or a typhoid, and many involuntary shudderings, carefully concealed, of course, from the patient, have to be overcome as the "skin case" walks into the out-patient room or is carried to his bed in the ward.

Why this repulsion of feeling? I do not think it is due to fear, for most nurses are never "afraid" of any disease, but simply to prejudice; and this can be speedily conquered. Personally, I do not mind touching or passing my hand over any skin eruption, and no nurse need fear doing the same. After contact with a contagious disease like scabies, or soiling the fingers with the discharge from ulcers, blebs or pustules, the hands should be immediately immersed in a 1 in 1,000 solution of the perchloride or biniodide of mercury, or a 2 per cent. lysol solution for one minute, and then washed with an antiseptic soap.

Every skin disease is not "catching," and there are only a very few which might really be termed loathsome. Such cases are not usually admitted into general hospitals, but even with these there will be no danger to the nurse of infection if the above precautions are carried out.

It should be borne in mind that sufferers from skin troubles are often extremely sensitive about their complaints, and they are quick to observe any suspicion of shrinking or unwillingness on the part of the nurse. The mental pain thus unsuspectingly produced adds tenfold to the physical distress caused by the disease.

*Acute eczema* is one of the commonest affections of the skin seen in hospital wards. In this, as in most cutaneous disorders, never forget that the patient is more or less ill in himself. The urine must be carefully collected and a specimen saved as soon as possible after admission. The nurse may have to test this herself, when the specific gravity and the presence of albumin or sugar must be specially noted.

Local treatment generally consists in the application of ointments and lotions. The former must be evenly spread upon the smooth side of the lint, if that material be used, though for the sake of economy fine linen is often employed. This is not nearly so good, as the ointment quickly soaks through the linen and ultimately through the bandages also, thus converting what might be a comparatively clean dressing into a saturated, greasy, unsightly and decidedly uncomfortable mess of rags. Do not cover up healthy skin with ointment, but cut the piece of lint the exact shape of the eczematous area. Only one limb or region of the body should be exposed at a time, and everything must be in readiness by the bedside before the final dressing is removed. Carefully bathe off the old ointment and any discharge which may have accumulated with the lotion ordered before applying the fresh.

In young children and infants with eczema all over the body and face, it is a good plan to cut out a sort of combination "mask" and jacket of lint, in one piece, fastened with tapes at the side. The hands will usually require tying up in wool during the night to prevent scratching.

The question of getting rid of crusts and scabs is an important one, and in district nursing in out-of-the-way places you may then have to act upon your own responsibility. If the crusts are separate and not very numerous, they may be covered with small pieces of clean linen rag soaked in a little carbolised oil, or even plain olive oil, for half a day. The scab will then be sufficiently loosened for you to bathe it off with tepid water without causing pain. For the more confluent crusting, as is often seen upon the heads of infants, the best application is a boracic starch poultice. The starch is made with a 2 per cent. boric acid solution to the consistence of a soft jelly. Apply this cold, or only just tepid, upon cotton-wool, and do not cover with jaconet or gutta-percha tissue. This may be renewed daily for three days, when the scalp will then be free from crusts and prepared for ointment dressings. The golden rule in all skin affections accompanied by crusting and scabbing is, "First remove the crusts, then apply the ointment." On no account is ointment to be laid on over a scab.

When acute eczema "weeps" it exudes a watery, albuminous fluid. This stiffens linen, so that if the ointment becomes dry the dressing will stick to the inflamed skin beneath. In such a case it is better to do the same as in burns, and to gently soak off the adherent lint in tepid bran-water. This variety of the disease is sometimes met with nearly all over the body, but is most common in the leg. It is called *eczema madidans.*

You will sometimes see besides the little vesicles and scabs characteristic of eczema small boils on adjacent parts of the skin. This means that the germs of common suppuration have been busy at work, and have obtained a foothold in the epidermis. How have they got in? Possibly the skin has been infected during the dressing. Never forget that a "raw" skin, such as is seen in acute inflammations or catarrhs like eczema, can absorb septic organisms quite as readily as an open wound, hence the absolute necessity for strict surgical cleanliness of the hands when touching or bathing the affected parts.

A word as to soaps. In hospital wards the doctor will, of course, order what kind of soap, if any, is to be used in a given case. As a general rule all kinds are harmful in the acuter stages of eczema. Then bran, which is very soothing, or fine oatmeal, sewn up in a small muslin bag, can be used for purposes of ablution. I need hardly say that many of the widely advertised toilet soaps which profess to "cure all skin diseases" are most injurious, though patients in their despair catch at them as the drowning man does a straw; indeed, I have known them to actually produce forms of eczema in predisposed individuals.

There is a variety of eczema occurring in the groins and perinæum of young infants, known as *erythema intertrigo.* Sometimes it is very severe in neglected children. Strict cleanliness is called for in these cases. A favourite application is a dusting powder composed of boric acid, zinc oxide, and starch sprinkled pretty thickly over the parts after cleansing with bran-water or the lotion ordered and wiping *dry.* A little cotton-wool, renewed as often as may be necessary, may with advantage be dusted with the powder and laid in the folds and creases of the parts.

Some diseases of the skin are characterised by the appearance of blebs or bullæ, as they are termed, which may be filled with clear serum or even blood. *Pemphigus*, an affection of this nature, attacks young children who are nearly always admitted as in-patients on account of its frequently fatal tendency. It is a good rule when dressing cases which have large blisters to gently snip the bleb at its lowest point, allow the contained fluid to run out, and then cover the collapsed bulla with the ointment dressing. Never cut the shrivelled wall of the blister away, as by so doing you deprive the raw layer of epidermis beneath of its natural protection. It will come away gradually in time. Remember that Nature will never be hurried, and if she is forced she will retaliate by retarding the healing process.

(*To be continued.*)

### The American Society of Training Schools for Nurses.

BY A MEMBER.

The tenth annual convention of the American Society of Superintendents of Training Schools for Nurses, which was held in Pittsburgh on October 7th, 8th, and 9th, was both stirring and interesting. It was preceded by a convention of the Pennsylvania State Nurses' Association, the members of which remained by invitation to attend some of the sessions, so with a good representative number of the society itself a large body was convened.

The meetings were held in the Shenley Hotel, where many of the visiting members were staying.

The first meeting was called to order at 9.30 A.M. on Wednesday, October 7th, with the President, Miss Ida F. Giles, in the chair. The opening prayer by the Rev. Bishop Whithead was followed by addresses of welcome from the Rev. Maitland Alexander, President of the Board of Trustees of the Allegheny General Hospital, and Dr. J. H. McClelland, chief surgeon of the Homœopathic Hospital. Responses to these addresses were made by Miss Snively, of the Toronto General Hospital, and Miss Maxwell, of the Presbyterian Hospital, New York.

Reports from the various officers and committees were then submitted, after which the President gave a short address, mentioning many things which had been accomplished by the Society, and urging still greater efforts in the future.

### Recreation for Nurses.

Papers were next read upon the subject of the study of current events as a means of recreation for nurses, by Miss Jane Delano of Bellevue Hospital, New York, and Miss Jennie Cottle, of Minnequa Hospital, Pueblo.

A short discussion followed these papers, and it seemed to be the consensus of opinion that classes for such study should exist in all training schools, and could be so arranged and conducted as to be extremely interesting and often entertaining. At the subsequent discussion it was mentioned that there were golf links for the nurses at the Minnequa Hospital and also saddle horses for their use.

The second session was called on Thursday morning at 9.30, and after the report of council twenty-three candidates were elected to membership.

The appointment of the Nominating Committee and the report of auditors followed. Miss Nutting called the Society's attention to the financial deficit and consequent impossibility of offering further assistance to the maintenance of the Department of Hospital Economics at Teachers' College, Columbia University, New York. A report from the Publication Committee showed a debt for publishing the transactions of the Nursing Congress at Buffalo, copies of which had not all been sold.

A report from the Superintendent of Army Nursing, Mrs. Dita Kinney, told of the development of that branch of the work, its improvements, and general condition at present.

### Education of Nurses.

Miss Nutting presented to the Society a statement concerning advances made along educational lines relating to nurses' training, conspicuous among these being the establishment of courses of study and training in Domestic Economy and other subjects, preparatory to hospital work, in Pratt Institute, Brooklyn, N.Y., and Drexel Institute, Philadelphia, and some others throughout the country. Also a change in the preparatory course at Simon's College, Boston, Mass., all being quite significant of the growing interest and recognition of the true importance of nursing in other educational institutions. Miss Nutting suggested that a Committee on Education be created, which should follow up the improved methods of instruction adopted in various schools of nursing or in preparation for such work, and all movements which would tend to establish better and more thorough education for nurses, and keep the Society informed on these matters. Later the committee was formed.

### Nurses' Salaries.

As the non-payment system, in operation in many schools, bears so directly upon this subject, it was introduced as a topic of discussion, and several superintendents who had tried both plans—that of giving an allowance of perhaps ten dollars per month and allowing the pupils to furnish their own uniforms and text-books, or of doing away with the allowance and supplying these articles at the hospital's expense, gave their comparative experiences. It was shown that a nurse's expenses are about one-third of such an allowance, and it was urged that it would be a wiser expenditure of this really large sum which remains after supplying uniforms, etc., to use it in providing salaries for more instructors, better facilities for carrying on the general work of the school, and in maintaining a larger staff of pupils, thereby shortening the hours of duty and favouring broader education, than to have it wasted, as far as the school as an institution is concerned, in spending-money for the individual.

Miss Burdett's paper giving a detailed description of "The New Lying-in Hospital, New York," was read by Miss Nevins. It dealt particularly with the heating, lighting, and ventilation of this beautiful new building which stands as a model of a modern hospital. Photographs of different parts of the hospital and mechanical contrivances in operation were handed about, giving still clearer ideas of the plans and fittings.

### Hospital Construction.

Miss Goodrich's paper on "Some Common Points of Weakness in Hospital Construction" showed how frequently hospital equipments which would greatly facilitate the work of the nurses and conduce to more perfect sanitation, were omitted, and also the inconvenient situation of many work- or store-rooms in relation to the wards themselves. Miss Goodrich suggested that if the superintendents of nurses were more often consulted by those entrusted with the construction of hospital buildings, these difficulties might often be obviated.

The reading of Miss McKechmie's paper on "What has been Done in the Way of Legislation," was followed by the formation of a committee to frame a standard of education which should be recognised as presenting to applicants for State registration the minimum requirements for eligibility, specially with regard to—

1. Entrance requirements to schools of nursing with definite minimum requirements.

2. A definite course of study with minimum of subjects, time to be devoted to theory and practice, and minimum length of course of training.

### New Officers.

The election of the following new officers then took place:—

*President*—Miss Georgia M. Nevins, Garfield Hospital, Washington, D.C.

*First Vice-President*—Miss Ida F. Giles, Homœopathic Hospital, Pittsburgh.

*Second Vice-President*—Miss Jennie Cottle, Minnequa Hospital, Pueblo, Col.

*Secretary*—Miss M. A. Nutting, Johns Hopkins Hospital, Baltimore.

*Treasurer*—Miss Anna L. Alline, Teachers' College, Columbia University, New York.

*Councillors*—Miss Sophia Palmer, Rochester, New York; Miss Isabel McIsaac, Presbyterian Hospital, Chicago.

*Auditors*—Miss Mary A. Samuel, Roosevelt Hospital, New York.

Miss Palmer next gave a brief statement concerning the *Journal of Nursing*, reminding the Society that the journal was its vocal organ and needed the constant support and patronage of the members.

"The Teaching of Hygiene in Theory and Practice in Training Schools" was the subject of Miss McIsaac's paper, and the author pointed out some of the striking inconsistencies existing in the practical application of this branch of teaching.

Miss Nutting read Miss Dock's paper on "The Power and Responsibility of this Society in Public Action." Some of the measures and plans which Miss Dock advocated had been discussed and set in motion in the previous day's proceedings, notably the establishment of a committee on legislation, and resolutions protesting against correspondence schools.

MORE ABOUT HOSPITAL CONSTRUCTION.

An extremely interesting talk on "Modern Hospital Construction," by Mr. Frank Miles Day, the well-known architect of Philadelphia, excited perhaps more discussion than any paper of the morning, since it dealt with a subject of much interest to nurses and one of great practical importance. As Miss Goodrich's paper was still fresh in everyone's mind, there were many questions raised which Mr. Day answered so satisfactorily that it was felt that the paper and subsequent discussion were most helpful.

Later, a resolution expressing regret at the loss of Miss Hutchinson, the much loved superintendent of nurses at the Massachussets Homœopathic Hospital, whose recent death was deeply regretted by the members of the Society, was passed.

NEXT YEAR'S CONVENTION.

It was announced that the next convention would be held at Washington in January 1905. Miss Nevins, the new president, was introduced, and after making a few appreciative remarks and extending a cordial invitation to all to attend the convention at which she would act as hostess, the meeting was adjourned, and this concluded all matters of a formal nature, excepting a Council meeting which was held in the evening.

SOCIAL FESTIVITIES.

The afternoons and evenings were devoted to enjoying the gracious hospitality which characterised the entire series of meetings and receptions—the Pittsburgh hostesses discharging their duties in a charming manner.

A luncheon on the first day at the Shenley Hotel was followed by an afternoon of sightseeing, and in the evening a reception afforded strangers an opportunity of learning something more of their fellow workers.

EXHIBITION OF NEW NURSING APPLIANCES.

The Society was entertained the second day at luncheon at the Western Pennsylvania Hospital, after which the following nursing appliances were demonstrated and explained by nurses from the schools represented:—Miss Goodrich, of the New York Hospital, showed a very convenient and safe arrangement of the articles necessary for giving hypodermic injections; an original method of preparing drains in tubes, leaving strips of muslin hanging outside the stoppers to indicate the width of the drain to which they were attached, and by means of which the drains might be easily removed from the tube, and various electrical appliances, poultices, and bed-warmers which had been satisfactorily used under her observation. She also showed a small kettle, with an alcohol lamp attached, which, when operating, gave off formaldehyde fumes, and had been found of service in deodorising rooms and disinfecting small cabinets containing instruments, and of great relief to patients suffering from coryza. Miss Morris explained and applied some unique abdominal binders from the Boston City Hospital; also some chest swathes, and a very excellent restraining jacket for use in nursing restless children. She likewise showed an efficacious method of applying a restraining sheet, and a simple and secure head bandage.

IMPROVEMENT IN BATHING APPLIANCES.

Miss Van Blarcom demonstrated the appliances from the Johns Hopkins Hospital, among them an improved stretcher for lifting typhoid patients to and from their tub baths; a hammock used for bathing such children in the Orthopædic wards as may not be put into water because of plaster or other dressings; a sweat-bath apparatus, consisting of a stove-pipe elbow heavily covered with asbestos and fastened to a tripod and Bunsen burner, the latter being the source of heat; various kinds of extension stockings which had been found comfortable; stupes made of lamb's wool and flannel; an ice bag for use in pneumonia; an ice-bag cover which had been devised for satisfactorily applying small ice bags, and a steamer evolved from an ordinary tea kettle, for use in nursing diphtheria, croup, or other troubles relieved by a moist atmosphere. This last apparatus consisted of the main receptacle for water, with gauges, valves, and pipes so arranged as to make it a very safe and satisfactory appliance.

From the Presbyterian Hospital, New York, Miss Williams demonstrated the method employed in that institution for preparing for infusions of saline solutions, showing at the same time some clever metal devices for holding and protecting the rubber tubing, and using one flask as the source of two streams, all of them greatly facilitating this operation. She also showed a very dainty method of making and applying an ice poultice, using oiled silk for a bag of the desired size and shape, with the edges seamed with adhesive plaster to prevent leakage as the ice melted. Some carefully prepared devices for application of splints and padding in Colles' fracture were also inspected with great interest.

These demonstrations, a novel feature of the programme, were held in the amphitheatre, which was well filled, and the afternoon proved to be most profitable.

FINAL BANQUET.

A banquet at the Shenley Hotel in the evening was the crowning social event, and the ready responses to the toasts were quite worthy of the representatives who earlier in the day had discussed matters of greater moment.

Still further inspection of this city, the site of some of our greatest industries, occupied part of the last afternoon, which terminated in an informal reception at the Homœopathic Hospital, given by the Trustees and Ladies' Association, where a tour of the hospital, followed by music and refreshments, closed the convention which so many forces had conspired to make memorable.

### Where to Go.

BIRMINGHAM AND MIDLAND HOSPITAL FOR WOMEN (out-patient department).—Tuesday, November 3rd, 7.30 P.M. Entertainment by St. James's, Edgbaston, Minstrel Troupe, in aid of the new in-patient department.

### The Prevention of Bed-Sores.

### EXAMINATION QUESTIONS FOR NURSES.

The question was as follows:—State what precautions you would take for the prevention of bed-sores.

### The Successful Papers.

The character of the answers generally this month is very good. "Jersey" gains the first prize, because hers is the best all round answer. She mentions what is too often overlooked by the nurse the fact that there can be no hard and fast rule with regard to preventive measures, because what is excellent for one skin is almost poison to another. Also she very sensibly dwells on the fact that friction is far more important than the lubricant used. "Nurse D.," who gains honourable mention, would have gained the first prize for her very excellent paper, but, alas! she recommends painting a tender place with collodion. This used to be a favourite practice, but I thought it had passed away from our midst. It is a most pernicious thing. It wrinkles and draws at the edges, thereby threatening to produce what it is supposed to heal, and, what is worse, conceals with its waterproof shininess whatever evil may be subtly going on underneath. It is a pleasure to award the second prize to a male nurse, W. B. He gives no pseudonym. He wisely speaks of using ointment if the skin is dry, instead of confining the treatment to spirit of some kind.

### First Prize.

Cleanliness, absolute dryness, and removal of pressure, are three of the chief preventives of bed-sores, as well as a smooth draw-sheet. It is not only on the lower part of the back that we must look for bed-sores, the shoulders, hips, elbows, heels, knees, are all liable to them. The nurse must constantly be on the look-out for sores on any of these places.

Bed-sores may be prevented by, when possible, changing the position of the patient from time to time, easing him on to his side, with a pillow at his back, for support. If the patient cannot be turned, then a water-bed, water-pillow, or air cushion will relieve the pressure. Failing any of these, a round pad, hollow in the centre, made of wool and tow and covered with old linen or a bandage, will answer the purpose. Heel pads are useful to remove pressure from the heels.

When a patient is obliged to lie flat, the back and other parts should be done three times a day. There are many things used to harden the skin and prevent sores, but the nurse must find out what suits her patient's skin the best.

Brandy or brandy and glycerine, methylated spirit, zinc ointment, triple ointment, lanoline, zinc and boracic powder, are all good in their way, but whatever is used, the great thing is to rub it into the skin well, it is useless dabbing it on and then leaving it. Before using any of the above, the parts should be well washed and thoroughly dried. Common yellow soap is better than any fancy sort.

In cases of paraplegia there is a great tendency to bed-sores, and the nurse will have to constantly examine the patient to see that the skin shows no sign of going.

A nurse cannot be too careful with respect to bed-sores, for what may appear to be a wee speck, may, if not taken in time, be the commencement of a bed sore that will take months to heal, and be a constant reproach to the nurse.

Jersey.

### Second Prize.

In any case of illness, and especially where a long confinement to bed is necessary, bed-sores should be carefully guarded against. Attention to the following points should help to prevent their appearance:

1. Attention to the bed and clothing. A feather bed should be discarded, and if possible a water or an air bed obtained, as they greatly diminish the tendency to bed-sores. There must be no creases in the sheets or patient's clothing. No crumbs, etc., must be allowed to get into the bed, for the smallest crumb is likely to be the cause of an ugly bed-sore.

2. Perfect cleanliness. The patient must be changed immediately there is any necessity, and the skin carefully washed with soap and water, then gently but thoroughly dried with a soft towel. If there is any tendency to perspiration or moisture of the skin, this must be seen to frequently, while if the skin is very dry some oily or greasy application may be used. The importance of perfect cleanliness in the prevention of bed-sores cannot be too greatly appreciated.

3. Relieving pressure by change of position or by cushions, etc. The nurse must remember that frequently the patient can give no information as to when a change of posture is necessary, he should be moved regularly and methodically, the skin being carefully looked to each time the position is altered, and especial note taken of the localities where bed-sores are apt to form, viz. the shoulder, sacrum, elbows, knees, ankles, and heels. If pressure seems to be telling on any spot, then some arrangement must be made to relieve it. An air or water-cushion will be found useful, sometimes better results are obtained by rings of soft material cut to fit the part to which they are to be applied and stuffed with cotton wool, or sheepswool carefully teased out is to be highly recommended, a low cushion placed under the legs for short periods will relieve the heels.

4. Applications to the skin. The nurse will of course inquire whether the physician in charge of the case prefers any particular local application. After the parts abovementioned are washed, there should be gently rubbed into the skin some spirit, either methylated spirit or pure spirits of wine, or whisky or brandy may be used. When the spirits have dried the skin should be dusted with starch or oxide of zinc powder, a quantity is not to be used, but only enough to insure smoothness of the skin. The gentle rubbing restores the circulation and the spirits tend to harden the skin. If the case has a tendency to be wet (as in incontinence) some oil or zinc ointment should be used for the skin. A nurse should always remember that a bed-sore is an impediment to the patient's progress, and may result in death from exhaustion or pyæmia.

W. B. (Male Nurse).

### Honourable Mention.

The number of satisfactory answers this month is so large that honourable mention has been granted to six competitors. "Nurse D.," "Marguerite," who sends an admirable answer, "Surrey," "R. T. R.," who is also a male nurse, "Belinda," and "Nurse Rose."

### Chief Defects of the Papers.

The tendency of the larger number is to be too much governed by red tape; 19 out of every 20 nurses say the same thing—rub with methylated spirit and dust with boracic powder—all very well as far as it goes, but how about the aged whose skin is already as dry as parchment, or those who have a tendency to eczema, to whom in many cases boracic acid in any form acts like a bunch of nettles! Moreover, it is frequently a grievous mistake to dust with powder at all, especially with skins which perspire much, the effect of such skin action being to make a paste of the powder and roll it into almost invisible balls which irritate intolerably and adhere to the fine hairs often found on the skin. With this process, apparently beloved of most nurses, the seeds are laid for a flourishing bed sore.

Next week we shall have the interest of seeing what our fellow nurses across the seas have to contribute to our information on the subject.

### Question for November.

If called upon in district nursing or otherwise to arrange the diet of a person suffering from an exhausting disease such as consumption, and if that person belonged to a labourer's family whose wages did not exceed 18s. or thereabouts per week, what should you advise as the cheapest and most nourishing scale of diet?—The Examiner.

Rules.

The competition is open to all. Answers must not exceed 500 words, and must be written on one side of the paper only. The pseudonym, as well as the proper name and address, must be written on the *same* paper, and *not on a separate sheet.* Papers may be sent in for fifteen days only from the day of the publication of the question. All illustrations strictly prohibited. Failure to comply with these rules will disqualify the candidate for competition. Prizes will be awarded for the two best answers. Papers to be sent to "The Editor," with "Examination" written on the left-hand corner of the envelope.

N.B.—The decision of the examiner is final, and no correspondence on the subject can be entertained.

In addition to two prizes honourable mention cards will be awarded to those who have sent in exceptionally good papers.

### Everybody's Opinion.

### THE ANGLO-AMERICAN NURSING HOME AT ROME.

"An Onlooker" writes: To one who, though not a resident, knows Rome and its doings, "A Nurse's" letter in your issue of October 17th raises the following questions:—Is it customary to leave the vindication and defence of an institution in the hands of one of the staff? What sort of a nurse is she who could term an attack of typhoid fever and its concomitant evils a "mole hill?" How is one of the staff as conversant with the affairs of the matron's office as "A Nurse" would seem to be? The supply of nurses may not fail, but there is always the possibility that quantity may be substituted for quality, and the hospital from which a nurse graduates is the most important item in the valuation of her qualifications. This is surely a matter for the committee and matron to speak upon, to prove that the statements most categorically made by "An English Nurse" in The Hospital of September 5th, 1903, are not in accordance with facts, which is what "A Nurse" most unmistakably wishes to convey to her readers.

"Another English Nurse" writes: As one of the former nursing staff of the Anglo-American Home in Rome during a previous season, I venture to write to you regarding the letter of "A Nurse" in your issue of October 17th. Doubtless the committee and matron will be very pleased to read it, since for three whole years they have been trying to get nurses to return, and seem at last to have succeeded. The fact remains that the whole of the previous season's nursing staff protested against their treatment by the matron. "A Nurse" knows this and also that the committee did not seriously attempt to investigate their case. What has happened once may happen again, though the matron may have learnt a partial lesson. A wrong has been done and the committee have shown that they do not intend to listen to any complaint against the management of the home: there lies the danger, a committee who "care for none of these things," a foreign country, and a matron who knows her committee. Abuse is not argument, and it seems to me that "A Nurse" can, of her own knowledge, and apart from what may have been suggested to her, know absolutely nothing about the illness of an "English Nurse," since the latter, according to her own letter, never returned to the home after her attack of typhoid. I do not think, therefore, that she is justified in saying that mountains have been made out of molehills, or that an "English Nurse" has stated what was untrue. She declares that she is speaking for the "rest of the staff," but she seems in reality to be speaking for the matron, whose correspondence she is apparently well acquainted with and even for the home itself! Why do not the matron and the committee defend themselves?

"Sister" writes: By the merest chance The Hospital of September 5th, 1903, was shown to me the other day, with the remark, "Is that kind of thing possible?" My reply was, that if the Anglo-American Home were still under the same management as in 1901–1902 it was not only possible but only what might be expected. Every nurse who reads "An English Nurse's" letter will, I am sure, be full of pity for her, more especially if the reader has been there herself. We were a specially healthy set, the season I happened to be connected with the Anglo-American Nursing Home, but one little circumstance showed us how much consideration we should receive should we fall ill, and we were positively in terror in case anything should happen to us. Nurse C. was on night duty in the home, and became suddenly ill, but managed to hold out till morning, when she went to bed, having been unable to eat anything all night. About 2 p.m. the nurse on day duty contrived to slip up to see her. No one had been near her, and she had had no food. The day nurse contrived to smuggle up some beef tea from the kitchen, which revived her very much. About 4 p.m. a lady friend of Nurse C.'s living in Rome came to see her. She knew that she had not been feeling quite well, so she asked the matron how she was. Her reply was, "Oh! much better, I have just taken her up some tea." She tried to dissuade the friend from going up, but she insisted, and was much interested to learn on reaching Nurse C.'s room that the matron had never been near her, and that she had never had any tea at all. This is just a tiny sample. It is really sad that what might be a delightful home for patients and nurses, should be spoken of as a money-grabbing institution.

### NURSES AS STEWARDESSES.

"W." writes: Do the ladies and trained nurses who are anxious to get a post as stewardess know what the real work of a stewardess is? Unless there is any special arrangement made, or someone happens to be ill, needing her attention while on the voyage, the regular duties would largely consist of sweeping and dusting, making the beds, of course, washing up the teacups and glasses, cleaning the silver, and waiting at each meal in the saloon. At the end of the journey, when the passengers have gone, there is all the thorough cleaning to see to, cushions, curtains to beat and brush, floors to scrub, furniture to clean, carpets to shake—though the cabin boy may do the latter work if he can be found—and a good many other things to do of a similar nature. Some ladies, too, would find it rather humbling, I think, to take money presents from those of their own class, and passengers are mostly very generous and give good gratuities when leaving. I am a nurse myself, but have also been a stewardess, so I know. Of course, to anyone who likes the sea and the work, and takes a pride in her own particular ship, as I do, and is a good sailor, the life is very pleasant in some ways.

### Presentations.

Stirling District Asylum, Larbert.—Miss Wise, whose appointment to the matronship of Craig House, Royal Asylum, Edinburgh, was announced recently, has been presented by Dr. Robertson on behalf of the staff of the Stirling District Asylum, Larbert, with a handsome gold watch and chain.

David Lewis Northern Hospital, Liverpool.—Miss Janet Burn Anderson, matron of the David Lewis Northern Hospital, Liverpool, for 16 years, having resigned her appointment as she is leaving this country in order to reside with an invalid sister in Australia, was the recipient of several gifts. Prior to her departure, the committee and honorary medical staff presented her with a silver-fitted travelling dressing case bearing an inscription, a gold bracelet with diamonds and sapphires, and a cheque. The nursing staff gave her a gold bracelet and pearl brooch, the resident doctors a silver purse, the domestics a writing case, the laundry staff a travelling clock, and the engineers and porters a purse. Miss Anderson also received further gifts from individual members of the household.

Ipswich Workhouse Infirmary.—Last week Nurse Grayston, who is leaving Ipswich Workhouse Infirmary in consequence of her approaching marriage, was presented by the master of the workhouse, Mr. C. Grayson, on behalf of the subscribers, with a tea-service purchased by the contributions of the nursing staff, workhouse officials, and the members of the Recreation Association, as a token of the esteem in which she is held. Dr. J. R. Staddon, medical officer, joined with the master in testifying to the admirable manner in which Miss Grayston had discharged her duties, and she made a suitable acknowledgement.

### TRAVEL NOTES AND QUERIES.

By our Travel Correspondent.

To Naples by Sea (Kathleen).—You can take the North German Lloyd Line, sailing from Southampton, first single from London £15, second £10; or the Orient Pacific Line from London itself, first single £15, second single £11. Sailings from London, November 6th–20th, December 4th–18th; sailings from Southampton, November 17th–30th, December 1st, etc. Return tickets are not issued, but if you come back within four months a reduction of 33 per cent. will be allowed off the homeward ticket, and if within six months, 20 per cent. Cheapest land passage *viâ* Newhaven and Dieppe, first single £10 10s., second single £7.

Hotels, etc., in Naples (Alpha).—You are choosing the most expensive season in Naples, but by arrangement for a prolonged stay reasonable terms may be had. Macpherson's Hotel and Pension Britannique start pension terms from 8 francs, which is 6s. 8d. per day; the Hôtel de la Riviera, on the Riviera di Chiaia, from 8 francs; the Hôtel de Russie, in the Strada Santa Lucia, from 7 francs; the Hôtel Roma, Strada Santa Lucia a Mare, from 6 francs. Then pensions: that of the Pension Müller, at 4 Via Partenope, at 5 francs per day, a general favourite, and Pension Pinto Storey, 3 Parco Margherita. Furnished apartments you must apply for on the spot. Your banker will tell you the agent's name and address, but unless you speak Italian well I should not recommend them.

Liverpool to Lisbon by Sea (F. M.).—You can go by the Booth Line, first-class single, £6; first return, £10. No second-class on this line. Sailings October 28th, November 9th, 19th and 28th. There is also the Pacific Steam Navigation Company—first-class single, £8; first return, £12; second-class single, £5. Return tickets, second-class, are issued at a reduction of 10 per cent. from the two single fares. Sailings October 29th, November 12th and 26th. I cannot tell you how long a return ticket lasts, but I think four months. If you write to Messrs. Cook and Sons, Ludgate Circus, E.C., and use my name, they will tell you. You would do wisely to get your tickets through them as they will tell you of the later sailings. If you go by sea it is necessary to have a passport, which you can get through Messrs. Cook without trouble—indeed in any case it is advisable, and the cost is very small. It is often a great help in claiming letters at the Poste Restante. Life in hotels is expensive, but you might put up at one of the following for a few days whilst looking about for a permanent resting place; the terms *en pension* will be from 6s. 6d. per day approximately, varying according to the position and size of your bedroom: Hôtel Durand, 71 Rua das Flores; Hôtel de l'Europe, 16 Rua do Carmo; and Hôtel Continental, 14 Largo de Sao Domingos. This last is the cheapest I have any knowledge of. I believe there are a few boarding-houses, but I do not know anything of them. The money is a puzzle. It is too involved for explanation here, but you will soon get accustomed to it, and the people are honest and will not take advantage of your ignorance. There is an imaginary coin called a "reis," five of which go to our farthing. Copper coins called "vintem" and "pataca" are valued respectively at 20 reis and 50 reis. Small silver coins, meio and testao, are useful, each one representing our 2½d. and 4½d. Very commonly used are the dollar and half-dollar, 4s. 2d. and 2s. 1d. The dollar is worth 1,000 reis. You will gain somewhat in your exchange, but not so much as in Spain. The exact rate of exchange I cannot tell you; moreover it is constantly varying. Tell me if I can help you further.

### Appointments.

Ashton-under-Lyne Union.—Miss Florence Tattersall has been appointed superintendent nurse. She was trained at the Royal Infirmary, Manchester, and was afterwards on the private nursing staff. For the last four-and-a-half years she has been charge nurse and theatre sister at the Chorlton Union Hospital, Manchester. She holds the L.O.S. certificate.

Bradford Poor-Law Sanatorium, Eastley.—Miss Rosetta Harris has been appointed matron. She was trained at King's College Hospital, and was afterwards charge nurse at the Hospital for Consumption, Ventnor, sister at Bethnal Green Infirmary, and night superintendent at Shoreditch Infirmary, London.

Charing Cross Hospital, London.—Miss G. Cassandom Henman has been appointed home sister. She was trained at St. Bartholomew's Hospital, London, where she has since been sister and night superintendent.

Halifax Union Poor Law Hospital.—Miss S. E. A. Kidson has been appointed matron. She was trained at St. Mary's Hospital, Paddington, where she was afterwards staff and charge nurse. She has since been sister and night superintendent at St. Saviour's Infirmary, East Dulwich, and senior assistant matron at Camberwell Infirmary, S.E.

Homœopathic Hospital, Birmingham.—Miss Marion A. Mann has been appointed night sister. She was trained at the Preston Royal Infirmary, and was subsequently attached to the private nursing staff of that institution. She has since been charge nurse at the Park Hospital, Hither Green, London.

Hospital for Epilepsy and Paralysis, Maida Vale.—Miss E. Keith has been appointed matron. She was trained at the Western Infirmary, Glasgow. She has since been assistant matron of the City of London Hospital for Diseases of the Chest, and matron of the Tunbridge Wells General Hospital.

Pontypool and District Hospital.—Miss Sarah Ann Todd has been appointed matron. She was trained at St. George's Hospital, London. She has since been on the staff of the South Wales Nursing Institute, Cardiff, and district nurse and dispenser at Retford Hospital and Dispensary.

St. Anne's Home, Herne Bay.—Miss Elizabeth Palmer has been appointed matron. She was trained at the Children's Hospital, Highgate, and at Guy's Hospital, and has since been night superintendent at Gore Farm Hospital, and in succession housekeeper and assistant matron at the Park Hospital, Hither Green.

St. Mary (Islington) Infirmary.—Miss K. E. Ogler has been appointed assistant matron. She was trained at Guy's Hospital, London. She has since been ward sister at the National Hospital, Queen's Square, London; theatre, and afterwards ward, sister at the Royal Portsmouth Hospital, temporary matron at Surbiton Cottage Hospital, and night superintendent at Shirley Warren Infirmary, Southampton.

South Shields Workhouse Infirmary.—Miss E. E. Ingram has been appointed superintendent nurse. She was trained at the Devon County Hospital, and has since been superintendent nurse at the Ipswich Union Infirmary.

Wells and District Cottage Hospital.—Miss Mary E. Smith has been appointed matron. She was trained at the Children's Hospital, Birmingham, and at Blackburn Infirmary. She has since been district nurse in Cheltenham and Gloucester, and Queen's Nurse in Manchester and Wells.

### Echoes from the Outside World.

### Movements of Royalty.

The King returned to London on Saturday afternoon on the conclusion of his visit to Lord and Lady Londonderry. The new Master of the Ceremonies appointed by his Majesty is Colonel Douglas Frederick Rawdon Dawson, a cousin of the present Earl of Dartrey, who has had a distinguished military career, is a Knight Commander of the Iron Crown of Austria, an Officer of the Legion of Honour, and a Companion of the Order of St. Michael and St. George.

The Queen also returned to London on Saturday, crossing from Calais to Dover while there was a high sea running. When the *Empress* arrived alongside Dover Pier her decks were soaked. The Queen was met by the King on her arrival at Victoria a quarter of an hour after the scheduled time, and as their Majesties crossed the platform together they were loudly cheered by a large crowd which had assembled outside the barrier.

### New Ambassador at Washington.

The King has approved of the appointment of Sir Henry Mortimer Durand as British Ambassador at Washington in succession to the late Sir Michael Herbert. Three years ago Sir Mortimer became Ambassador to Spain, and from 1894 to 1900 he was Minister at Teheran. He was called to the Bar in 1872, and took up his first appointment in Bengal in the following year. He was Assistant Magistrate and Collector for a short time, his next post being in the Foreign Department of the Government of India. In this department he manifested so much ability that in 1880 he became Under-Secretary and in 1881 Secretary. In 1893 he successfully acquitted himself in a mission to the Ameer of Afghanistan, and the agreement he signed with the Ameer before leaving Kabul is generally known as "the Durand Agreement." He is in his 54th year, celebrated his silver wedding three years ago, has a son in the 9th Lancers who was wounded at Kimberley, is an expert shot, and has written a fairly successful novel.

### Death of a Famous Historian.

The death of Mr. W. H. Lecky took place in London on Thursday afternoon last week. Mr. Lecky represented Dublin University in the House of Commons for a few years, but he was chiefly known as a great historian, and he was chosen by the King as one of the original members of the Order of Merit. He was a native of Dublin, was born in 1838, and, having passed through Cheltenham College, graduated at Trinity College. His first historical book was "The History of European Morals from Augustus to Charlemagne," and then came his principal works, "The History of England in the Eighteenth Century," and his "Democracy and Liberty." The remains of Mr. Lecky, whose widow is a Dutch lady, were cremated, and the funeral took place on Wednesday.

### Political Murder in London.

The President of the Armenian Refugee Society in London, M. Saghitiel Sagouni, was shot with a revolver on Monday night as he was entering the house in which he was living at Nunhead, and died shortly afterwards. It is believed that the murderer, who made good his escape under the cover of darkness, is connected with another Armenian Association which disapproves of the methods of the organisation to which the murdered man belonged.

### Lightning Speed for Trains.

Further trials have been made of the power of the electric trains in Germany. This time the Kaiser was not present, but the speed attained was even yet more wonderful than before. The experiments took place on the military line between Zossen and Marienfelde, and though amongst the spectators some requested to be allowed to ride in the carriage, with the exception of the engineers and half a dozen specialists—which included Dr. Wilhelm von Siemens—no one was allowed to experience the novel sensation of flying through the air. An onlooker thus describes the journey of the train:—"When the military horn had been blown as the signal of departure, the ground under the spectators' feet seemed to tremble as during an earthquake, a rumbling sound as of thunder was heard, and overhead a line of blue sparks rushed onwards, dazzling the eye. Suddenly a two-storey waggon came in sight, and as it flitted past something white could be descried inside, but it was impossible to determine whether it was a human face or a sheet of paper, so rapid was the passing. The next instant the waggon was merely a speck in the distance, whilst a faint rumbling sound was heard." The train made four trips and the last time the speed attained was 130 miles an hour!

### Society of British Artists.

At the Galleries in Suffolk Street, the Royal Society of British Artists opened its winter exhibition on Monday. There is nothing of conspicuous merit, but Sir Wyke Bayliss is as impressive and as reverential as usual in his representation of church architecture "Duflamme of Milan," and Mr. Sheard has a vigorous and interesting representation of the "School for Scandal." The scholars, however, are not in high life, they are women of the working classes, in a cottage, where they are drinking tea and as effectually taking away their neighbours' characters as if they had been chatting in miladi's boudoir. The malevolent enjoyment on some of the faces is, perhaps, a little too accentuated, but it is a clever picture and tells its story well. Amongst the portraits there is an admirable likeness of the King by Mr. Fuchs, and Mr. Cave Day's "Lady in White" must not be overlooked, whilst "Dr. Parker's First Sermon" has an interest of its own. Mr. Haité has painted a Jubilee picture which is worth noting, and amongst landscapes Mr. Talmage's "When Lingering Daylight Welcome's Night's Pale Queen," Mr. Rupert Burny's "Grey Day," and Mr. Walter Fowler's "A Country Lane" are deserving of mention.

### The Gaiety Theatre.

The new theatre built on the site of the old "Gaiety" opened on Monday night, and as early as five o'clock in the morning, before the daylight, the first "galleryite" arrived. By breakfast time he was joined by some ladies with camp-stools, and all through the day, notwithstanding the pelting rain, the crowd increased till, in the evening, a thousand persons were in the streets waiting admission. The theatre is both beautiful and luxurious, and the piece, "The Orchid,", is a musical play written specially to please the patrons of the new Gaiety, whose tastes have evidently not changed with the house itself. The libretto is by Messrs. Adrian Ross and Percy Greenbank; the music by Messrs. Ivan Caryll and Lionel Monckton, and the dances and the dresses are as fantastic and as dainty as usual. The incidents hinge upon the possession of a valuable plant which is always being lost and the troubles of a couple of couples who were married in the wrong pairs owing to the blunder of a foolish Registrar. There is much bright music and there are plenty of comic songs, the chorus being particularly in evidence all through the play. Miss Connie Ediss, Miss Gertie Millar, Miss Gabrielle Ray, as well as Mr. Edmund Payne, Mr. Fred Wright, jun., and Mr. George Grossmith, jun., are all in the cast and act and sing and dance as if the performance afforded them as much pleasure as their patrons. The King and Queen were present at the performance.

### For Reading to the Sick.

ALL SAINTS' DAY.

They are not dead, but sleeping:
Vex not them
With tears and lamentations in your sorrow;
Short is the time before the golden morrow
Shall shed its welcome beam—
The New Life's pathway in fresh glory steeping.

They are not dead, but sleeping:
Softly rest,
Ye dear departed, in your tranquil home,
Sleep on in peace, till your kind Lord shall come
And bear you in his breast
Far from the sounds of earthly grief and weeping.

*From the "Waiting Church."*

What do they do, those blessed beings of whom the text speaks? Whatever else they do, or do not do, this we are told they do—they worship. They satisfy, it would seem, in perfection, that mysterious instinct of devotion—that inborn craving to look upward and adore, which, let false philosophy say what it will, proves the most benighted idolater to be a man, and not a brute—a spirit, and not a merely natural being.

They have worshipped, and so are blest. They have hungered and thirsted after righteousness, and now they are filled. They have longed for, toiled for, it may be died for, the true, the beautiful, and the good; and now they can gaze upward at the perfect reality of that which they saw on earth, only as in a glass darkly, dimly, and afar; and can contemplate the utterly free, the utterly beautiful, and the utterly good in the character of God and the face of Jesus Christ. They entered while on earth into the mystery and the glory of self-sacrifice; and now they find their bliss in gazing on the one perfect and eternal Sacrifice, and rejoicing in the thought that it is the cause and ground of the whole universe, even the Lamb slain before the foundation of the world.

I say not that all things are clear to them. How can they be to any finite and created being? They, and indeed angels and archangels, must walk for ever by faith, and not by sight. But if there be mysteries in the universe still hidden from them, they know who has opened the sealed book of God's secret counsels, even the Lamb who is the Lion, and the Lion who is the Lamb; and therefore if all things are not clear to them, all things at least are bright, for they can trust that Lamb and His self-sacrifice. In Him and through Him, light will conquer darkness, Justice injustice, truth ignorance, order disorder, love hate; till God be all in all, and pain and sorrow and evil shall have been exterminated out of a world for which Christ stooped to die. Therefore they worship; and the very act of worship—understand it well—is that great reward in heaven which our Lord promised them.—*Charles Kingsley.*

No Angel comes to us to tell
Glad news of our beloved dead;
Nor at the old familiar board,
They sit among us breaking bread.
Three days we wait before the tomb,
Nay lifelong years; and yet no more,
For all our passionate tears, we find
The stone rolled backward from the door.
Yet are they risen as He is risen;
For no eternal loss we grieve,
Blessed are they who ask no sign,
And, never having seen, believe.

*Lewis Morris.*

### Notes and Queries.

**FOR REGULATIONS SEE PAGE 21.**

*Uniform.*

(39) I should be pleased to know if a nurse holding the Medico-Psychological certificate has a right to wear uniform?—*I. L.*

There are no restrictions as regards nurses' uniform.

*Cottage Hospitals.*

(40) *Mrs. M.* would be much obliged if the Editor would tell her where she could get a list of cottage hospitals in Scotland.

In "Burdett's Hospitals and Charities." The Manager, the Scientific Press, 28 Southampton Street, Strand, W.C., would supply the book. Price 5s.

*Maternity.*

(41) Will you kindly give me the address of lying-in hospitals in Manchester and their fees? I should like to know the cheapest; and if there is a hospital connected with the Salvation Army.—*Nurse C.*

The Manchester Maternity Hospital, 60 Upper Brook Street. Fees, midwifery, £15 15s.; pupil nurses, £11 11s. St. Mary's Hospital and the Manchester and Salford Lying-in Hospital and Dispensary, Quay Street. Maternity pupils pay a fee of £1 1s. a week, and are resident for at least six weeks. Any information about the Salvation Army may be obtained at their headquarters, 101 Queen Victoria Street, E.C.

*Far East.*

(42) I should be glad if you will kindly inform me which is the best way to obtain work in a missionary hospital either in China or India; and also if missionary societies pay salaries?—*M. E. O.*

Write to the Church Missionary Society, 14 and 16 Salisbury Square, E.C. Yes.

*Midwifery.*

(43) Will you kindly tell me where to go to be registered as a midwife? I have been in practice for 16 years, and hold certificates from the L O.S. and Queen Charlotte's.—*A. H.*

Apply to the Secretary, the Central Midwives Board, 6 Suffolk Street, Pall Mall, S.W.

*Lecturer.*

(44) Can you kindly tell me if it is possible for a nurse with two years' hospital training to become a lecturer under the London County Council?—*G. R. H.*

Apply the Secretary, Technical Education Board, London County Council, Spring Gardens, S.W.

*Hospital Training.*

(45) I am desirous of being trained as a nurse in a children's hospital. Can you give me any information as to the best hospital to enter as probationer?—*E. E. G.*

See "The Nursing Profession: How and Where to Train," published by the Scientific Press.

*Bombay.*

(46) Nurse S. would be very glad if the Editor would tell her (1) if there are any good hospitals in Bombay, and (2) also if there are any openings for private nurses.

1. The majority of nurses in the Bombay hospitals are Eurasians. A full list may be seen in Burdett's "Hospitals and Charities." 2. Unless you have some influence amongst medical men in Bombay, we should advise you not to start as a private nurse.

*Home.*

(47) Can you tell me of an institution where a lady of 65, who has been paralysed for four years, could be received? She has no control over her emotions. Her daughters could afford to pay a small sum towards her support.—*Marflo.*

The Home for Confirmed Invalids, 36 Aubert Park, Highbury, N., the Midland Home for Incurables, Leamington, or St. Peter's Home and Sisterhood, Mortimer Road, Kilburn, N.W., seem suitable for the case.

**Important Nursing Textbooks.**

"The Nursing Profession: How and where to Train." **2s. net; 2s. 4d. post free.**

"A Handbook for Nurses." (New Edition). **5s. net; 5s. 4d.** post free.

"Practical Guide to Surgical Bandaging and Dressings." By Wm. Johnson Smith, F.R.C.S. 2s. post free.

"The Nurses' Dictionary of Medical Terms and Nursing Treatment." By Honnor Morten. 2s. post free.

"The Human Body: its Personal Hygiene and Practical Physiology." By B. P. Colton. 5s. post free.

"Art of Feeding the Invalid." (Popular Edition). **1s. 6d. post free.**

"On Preparation for Operation in Private Houses. By Stanmore Bishop, F.R.C.S. 6d. post free.

